# Fast cross-staining alignment of gigapixel whole slide images with application to prostate cancer and breast cancer analysis

**DOI:** 10.1038/s41598-022-15962-5

**Published:** 2022-07-08

**Authors:** Ching-Wei Wang, Yu-Ching Lee, Muhammad-Adil Khalil, Kuan-Yu Lin, Cheng-Ping Yu, Huang-Chun Lien

**Affiliations:** 1 Graduate Institute of Applied Science and Technology, National Taiwan University of Science and Technology, Taipei, Taiwan; 2 Graduate Institute of Biomedical Engineering, National Taiwan University of Science and Technology, Taipei, Taiwan; 3 Department of Pathology, Tri-Service General Hospital, Taipei, Taiwan; 4 Institute of Pathology and Parasitology, National Defense Medical Center, Taipei, Taiwan; 5 Department of Pathology, National Taiwan University Hospital, Taipei, Taiwan; 6 Graduate Institute of Pathology, National Taiwan University, Taipei, Taiwan

**Keywords:** Biomedical engineering, Cancer

## Abstract

Joint analysis of multiple protein expressions and tissue morphology patterns is important for disease diagnosis, treatment planning, and drug development, requiring cross-staining alignment of multiple immunohistochemical and histopathological slides. However, cross-staining alignment of enormous gigapixel whole slide images (WSIs) at single cell precision is difficult. Apart from gigantic data dimensions of WSIs, there are large variations on the cell appearance and tissue morphology across different staining together with morphological deformations caused by slide preparation. The goal of this study is to build an image registration framework for cross-staining alignment of gigapixel WSIs of histopathological and immunohistochemical microscopic slides and assess its clinical applicability. To the authors’ best knowledge, this is the first study to perform real time fully automatic cross staining alignment of WSIs with 40× and 20× objective magnification. The proposed WSI registration framework consists of a rapid global image registration module, a real time interactive field of view (FOV) localization model and a real time propagated multi-level image registration module. In this study, the proposed method is evaluated on two kinds of cancer datasets from two hospitals using different digital scanners, including a dual staining breast cancer data set with 43 hematoxylin and eosin (H&E) WSIs and 43 immunohistochemical (IHC) CK(AE1/AE3) WSIs, and a triple staining prostate cancer data set containing 30 H&E WSIs, 30 IHC CK18 WSIs, and 30 IHC HMCK WSIs. In evaluation, the registration performance is measured by not only registration accuracy but also computational time. The results show that the proposed method achieves high accuracy of 0.833 ± 0.0674 for the triple-staining prostate cancer data set and 0.931 ± 0.0455 for the dual-staining breast cancer data set, respectively, and takes only 4.34 s per WSI registration on average. In addition, for 30.23% data, the proposed method takes less than 1 s for WSI registration. In comparison with the benchmark methods, the proposed method demonstrates superior performance in registration accuracy and computational time, which has great potentials for assisting medical doctors to identify cancerous tissues and determine the cancer stage in clinical practice.

## Introduction

Monitoring of multi-modal molecular and cellular interactions, tumor growth and tissue morphology is critical for cancer diagnosis, drug development and biological research and usually performed by analyzing multiple microscopic slides with various staining simultaneously. That is joint analysis of a hematoxylin and eosin (H&E) slide to assess the cellular morphology and multiple immunohistochemical (IHC) slides to monitor various protein expression patterns together at single-cell microscopic resolution^1^. In practice, H&E staining is adopted as a gold standard to analyze tissue morphology. On the other hand, IHC staining is widely utilized to detect antigens, namely proteins, in diagnosis of abnormal cells such as those found in cancerous tissues, enabling biologists and medical doctors to observe the distribution of proteins in different parts of biological tissue. In neuroscience, IHC allows scientists to closely inspect protein expressions within specific brain structures^2–4^. In drug development, IHC has been applied to test drug efficacy by monitoring certain protein expressions and analyzing the active levels of disease targets^5,6^. In clinical diagnosis, with the knowledge of tumor marker and IHC tools, medical professionals and scientists are able to diagnose tumors as benign or malignant, determine the cancer stage and identify the cell type and origin of a metastasis in order to find the site of the primary tumor.

**Figure 1:**
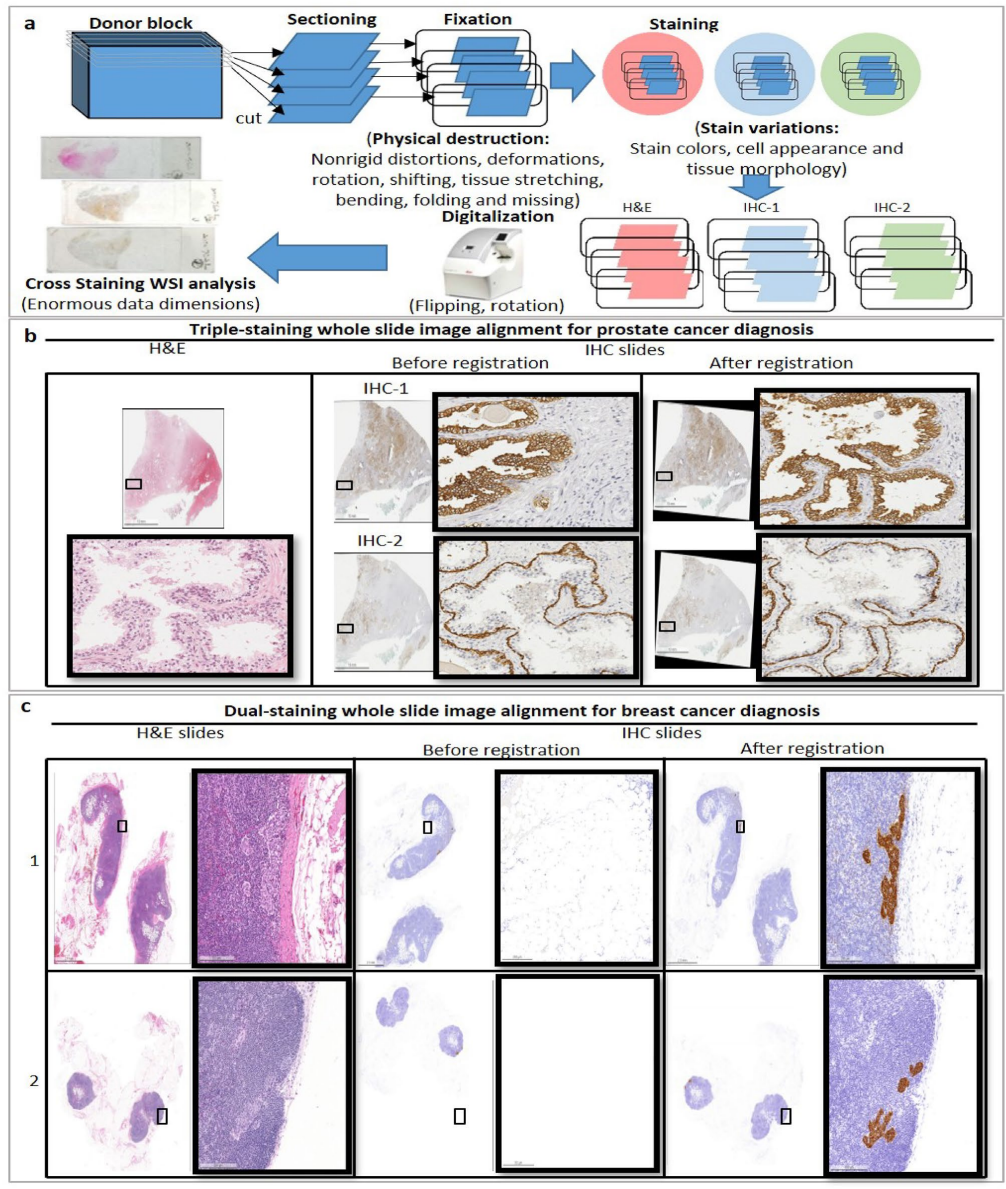
(**a**) Challenges caused by individual data preparation steps for cross staining WSI analysis. Sample results by the proposed method in (**b**) triple-staining WSI alignment of H&E, HMCK and CK18 slides for prostate cancer diagnosis and in (**c**) dual-staining WSI alignment of H&E and CK(AE1/AE3) slides for breast cancer diagnosis.

However, to align, compare, and quantify protein expressions of tissues of interest from multiple whole slide images (WSIs) with different stainings is difficult due to the enormous dimensions of gigapixel images to analyze, nonrigid distortions and deformations introduced during slide preparation and dissimilar cell appearance and tissue morphology across stainings. Figure 1a shows challenges induced from individual data preparation steps for cross staining WSI analysis in cancer diagnosis. Serial sections are firstly cut from a patient donor block and mounted onto glass slides, causing nonrigid deformations and distortions across slides. Secondly, slides are processed with various kinds of stainings. As each staining highlights specific substances in tissues, the image appearances of cells and tissue layouts across various stainings become distinctively dissimilar. Thirdly, slides are digitized into enormous gigabyte-sized WSIs at single-cell microscopic resolution, including a H&E WSI and multiple IHC WSIs to observe tissue morphology and various protein expression patterns together. Typically, WSIs are stored in a gigabyte-sized multi-resolution pyramid data structure from low to high magnification with a general size of around 100,000 $\times$ 200,000 pixels at the highest resolution level. In addition to the enormous dimensions of data to process, there are large variations on stain colors, cell appearance, and tissue morphology across WSIs.

Due to the advancement of computing power, many computer algorithms^7–10^ have been developed for automatic alignment of biological microscopic images. The bUnwarpJ tool by Arganda-Carreras et al.^7^ is developed for biological section alignment, allowing bi-directional image registration. Saalfeld et al. presented a least square based registration method using linear feature correspondences^8^ and an elastic registration approach based on non-linear block correspondences^9^. Wang et al. built a CwR method^10^, which contains a 3D alignment and validation model utilizing the b-spline deformation field. In this study, the above mentioned approaches^7–10^ are adopted as benchmark methods.

From the literature review, previously many studies^11–20^ performed image alignment by directly applying image transformation to entire images. Later, several approaches^21–26^ have been developed with a more efficient scheme such as hierarchical frameworks to deal with large microscopic datasets. Due to the advancement of imaging technology, image alignment is required in application to enormous gigabyte-sized images such as WSIs. The above-mentioned methods are however too memory costly and slow to be applied to WSI alignment. For dealing with the enormous size of WSIs, several tile-based approaches have been built. Roberts et al.^27^ presented a multi-resolution-block-matching-based nonrigid registration framework. Song et al.^28^ and Lotz et al.^29^ extended Roberts’s approach^27^ for tissue reconstruction of histological sections with different stains. Song et al. developed an unsupervised content classification method that produces multichannel probability images from images of different stains to increase the similarity of the two images and integrated the classification method into the multi-resolution-block-matching-based framework. Lotz et al.^29^ first computes nonlinear registration on low-resolution images to correct global large-scale deformations occurring in tissues, and the results of this nonlinear registration is then used as an initial guess for a patch-wise registration. In this study, the three tile-based approaches for WSI alignment^27–29^ are adopted as benchmark methods for run time analysis.

Prostate cancer is the second leading cause of cancer related death in men in the United States^30^. In 2018, prostate cancer has caused 358,989 deaths worldwide^31–33^. In diagnosis of invasive prostate carcinoma, it is vital to check if the basal cell layer is absent or not. Hence, a complete absence of staining in basal cell-associated markers is supportive of a malignant interpretation. The diagnostic and prognostic value of CK18 in prostate cancer and human tumor has been proved and described in^34–36^. In 2012, Weng et al.^34^ pointed out that CK18 is expressed in a variety of adult epithelial organs, plays a role in carcinogenesis and in combination with other markers as an important marker of therapeutic efficacy. In 2016, Yin et al.^35^ determined that the IHC CK18 expression in PCa tissues is inversely correlated with tumor grade in a statistically significant fashion ($p=0.028$) where tissue samples with higher tumor grades (Gleason score $\ge 7$) show gradually decreased CK18 intensity. In 2021, Menz et al.^36^ show that reduced CK18 immunostaining is linked to high UICC stage ($p=0.0010$), high Thoenes grade ($p=0.0086$) and advanced tumor stage ($p<0.0001$) based on 11952 tumor samples. Using IHC, High-Molecular-Weight Cytokeratin (HMCK) is a cytoplasmic marker that highlights intermediate cytokeratin (CK) filaments in glandular basal cells and is specific for basal cells in the prostate.

In clinical practice, manual cross-staining analysis of whole slides is conducted; in order to diagnose a sample as benign or malignant, medical doctors have to manually identify cancerous tissues first and then determine the tumour stage and grade of a patient with prostate cancer. As only the tissues with positive CK18 detected and within the gland with positive HMCK detected are determined as cancerous tissues, in order to identify cancerous tissues and determine the tumor stage and grade, medical doctors have to manually align three gigabyte-size microscopic WSIs of each patient and then subjectively quantify cancerous tissues of WSIs, to perform simultaneous analysis of an IHC HMCK slide, an IHC CK18 slide and a H&E slide. This is however difficult and poorly reproducible considering the enormous dimensions of gigapixel images to analyze, nonrigid distortions and deformations introduced during slide preparation and dissimilar cell appearance and tissue morphology across different staining. Automatic cross-staining alignment of WSIs enables medical experts and scientists to easily identify the cancerous tissues, quantify the amount of cancerous tissues and determine the stage and grade of a tumour. Figure 1b shows the automatic triple-staining result of prostate cancer sample by the proposed method.

Breast cancer is the most prevalent cancer in women and a major cause of mortality^37,38^. In prognosis of patients with breast cancer, the five-year survival rate is 90%, and ten-year is 83%. However, the survival rate is significantly worsened when breast cancer metastasizes^38,39^. In the case of localized breast cancer, the five-year survival rate is 99%, which drops to 85% in the case of regional (lymph node) metastases. Furthermore, the five-year survival rate is 26% in the case of distant metastases. Therefore, it is important to identify the metastases to provide adequate treatment and to improve the survival rate. The prognosis of breast cancer patients is mainly determined by the extent of the cancer evaluated using the TNM staging system that assesses the size of the tumour (T), the status of axillary lymph nodes (N) and the status of metastasis (M). The status of axillary lymph nodes could be estimated by evaluating the sentinel lymph node, which is the first lymph node to receive the afferent lymphatic drainage from the primary cancer and is thus the first node to be involved by metastasis. Therefore, patients whose sentinel lymph node is negative for breast cancer metastasis could be spared for more extensive axillary lymph node dissection. Alternatively, axillary lymph node dissection may be performed on breast cancer patients, particularly for patients with high suspicion of axillary lymph nodes metastasis.

Metastatic foci to lymph nodes have been classified into macrometastases (metastatic size greater than 2.0 mm), micrometastases (metastatic size greater than 0.2 mm, but none greater than 2.0 mm), and isolated tumour cells (ITCs, metastatic size no greater than 0.2 mm). It is difficult for human to identify ITCs due to their tiny size, particularly in cases with many axillary lymph nodes, leading to under-treatment of the patients. Furthermore, the routine examination of H&E slides of lymph nodes, particularly for axillary lymph node dissection, which may contain many lymph nodes, is time consuming and pathologists are at the risk of missing tiny metastatic foci. The MIRROR (Micrometastases and Isolated tumour cells: Relevant, Robust or Rubbish) trial emphasized the need for additional therapy in cases of invasive breast carcinoma with ITCs or micrometastases^40–43^, supporting the identification of ITCs or micrometastases is urgently needed in the management of breast cancer patients. IHC staining for cytokeratin on lymph node section could be used to detect ITCs. In this study, we illustrate automatic cross-staining alignment of WSIs of H&E and IHC CK(AE1/AE3) slides help screen all the lymph nodes and identify tiny metastatic foci to improve the accuracy of pathological assessment of lymph node as shown in Fig. 1c.

**Figure 2:**
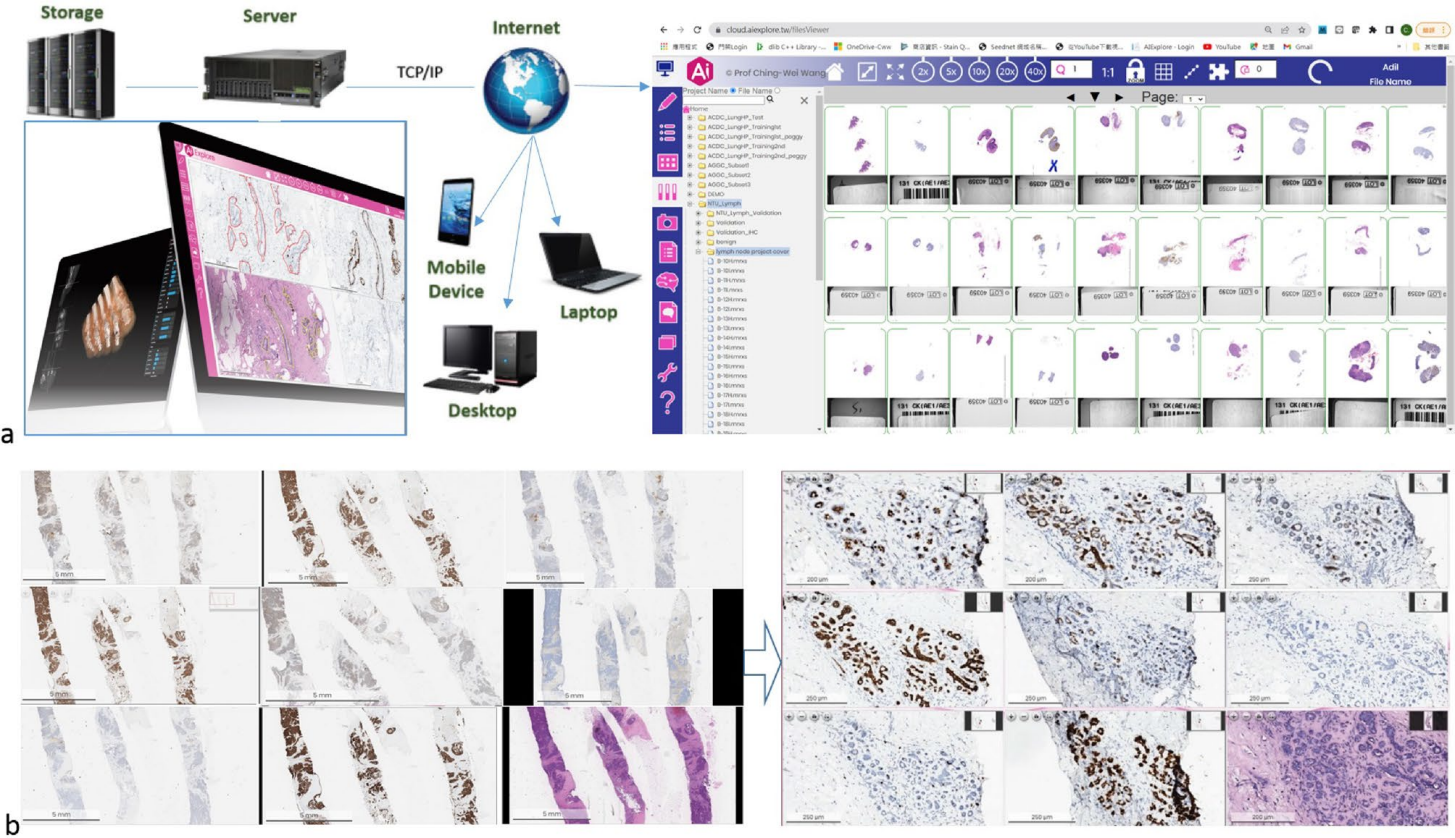
Flowchart in clinical usage for the proposed method in cross staining WSI analysis. (**a**) Medical experts access the WSI database on a web browser through their devices. (**b**) The proposed method performs real time registration and assists analysis of multiple WSIs simultaneously.

In this paper, we present a real time interactive fully automatic hierarchical registration framework that is able to perform cross-staining alignment of gigapixel WSIs of histopathological and immunohistochemical microscopic slides in real time and also overcome the major speed bottleneck for assisting medical doctors to identify cancerous tissues and determine the stage and grade of a tumor. The proposed hierarchical registration framework is described in detail in “Methods” section . The effectiveness and robustness of the proposed fully automatic hierarchical registration framework is validated using two datasets, including a dual staining breast cancer dataset with 43 H&E slides and 43 IHC CK(AE1/AE3) slides and a triple staining prostate cancer dataset with 30 H&E slides, 30 IHC CK18 slides, and 30 IHC HMCK slides. Figure 1b,c presents the automatic WSI alignment result of triple-staining prostate cancer samples and dual-staining breast cancer samples by the proposed method, respectively. Figure 2 illustrates the flowchart for the proposed method in cross staining WSI analysis in clinical usage. In addition, an online web-based system of the proposed method has been created for live demonstration of real time cross staining alignment of whole slide images. Please see the supplementary video at https://www.youtube.com/watch?v=0Uc6-s_ClIg&ab_channel=ProfChing-WeiWang.

## Data and results

### Materials

In this study, two datasets of WSIs were built and collected from two different hospitals, including a dual staining breast cancer dataset with 43 H&E slides and 43 IHC CK(AE1/AE3) slides collected from National Taiwan University Hospital, Taipei, Taiwan with an ethical approval (NTUH-REC 201810082RINB) and a triple staining prostate cancer dataset with 30 H&E slides, 30 IHC CK18 slides, and 30 IHC HMCK slides collected from Tri-Service General Hospital, Taipei, Taiwan, with an ethical approval (TSGHIRBI-107-05-171). The slides were generated under routine sample preparation procedures. Firstly, tissues are fixed in 10% buffered formalin and embedded in paraffin. Next, serial sections were cut (4 μm) using Leica RM2155 and then stained with H&E and associated IHC stains. In digitization, the prostate cancer slides were scanned using Leica AT Turbo scanner (Leica. Wetzlar Germany) at 40× objective magnification and the breast cancer slides were scanned using 3DHISTECH Pannoramic SCAN II scanner (3DHISTECH Kft. Budapest Hungary) at 20× objective magnification. We summarized the information of the two experimental data sets in Table 1.

**Table 1:** Information of the experimental datasets.

Datasets	Staining	WSIs	Size (${\text {mm}}^2$)	Size (pixels)	Scanner (File format)/Hospital	Obj. Mag.
Prostate Cancer	H&E	30	$29.02\times 22.93$	$117095 \times 92537$	Leica AT Turbo (.svs)/Tri-service General Hospital, Taiwan	40×
	HMCK	30	$28.23\times 22.09$	$113921 \times 88140$		
	CK18	30	$28.94\times 21.85$	$116777 \times 88159$		
Breast Cancer	H&E	43	$25.11\times 50.63$	$113501\times 228816$	3DHISTECH Pannoramic Scan II (.mrxs)/National Taiwan University Hospital	20×
	CK (AE1/AE3)	43	$25.11\times 50.63$	$113501\times 228816$		

### Results

In evaluation, three experiments were performed, including an experiment on cross-staining alignment on triple staining prostate cancer slides (see “Quantitative evaluation on the triple-staining prostate cancer dataset” section ), an experiment on cross-staining alignment on dual staining breast cancer slides (see “Quantitative evaluation on the dual-staining breast cancer dataset” section ) and an experiment on run time analysis (see “Run time analysis” section ). The first evaluation was conducted on 30 sets of triple-staining prostate cancer samples, including H&E slides and IHC slides with two kinds of antigens, CK18 and HMCK, and we compare the proposed method with four image registration techniques for biological microscopic image alignment, including bUnwarpJ^7^, LeastSquares^8^, Elastic^9^ and CwR^10^. The second evaluation was performed on 43 pairs of dual-staining breast cancer slides, including 43 H&E slides and 43 IHC slides with CK(AE1/AE3) antigen, and we further compare the proposed method with the best benchmark approach performed in the first experiment, i.e bUnwarpJ^7^. The third experiment was performed to produce run time analysis on the 43 pairs of dual-staining breast cancer slides. We compare the computational time in WSI registration of the proposed method and the best benchmark approach performed in the first experiment, i.e bUnwarpJ^7^, Roberts et al.^27^, Song et al.^28^ and Lotz et al.^29^. All statistical analysis was performed using SPSS software^44^, and all experiments were performed on a workstation with an Intel Xeon Gold 6134 CPU processor and 64GB RAM.

**Figure 3:**
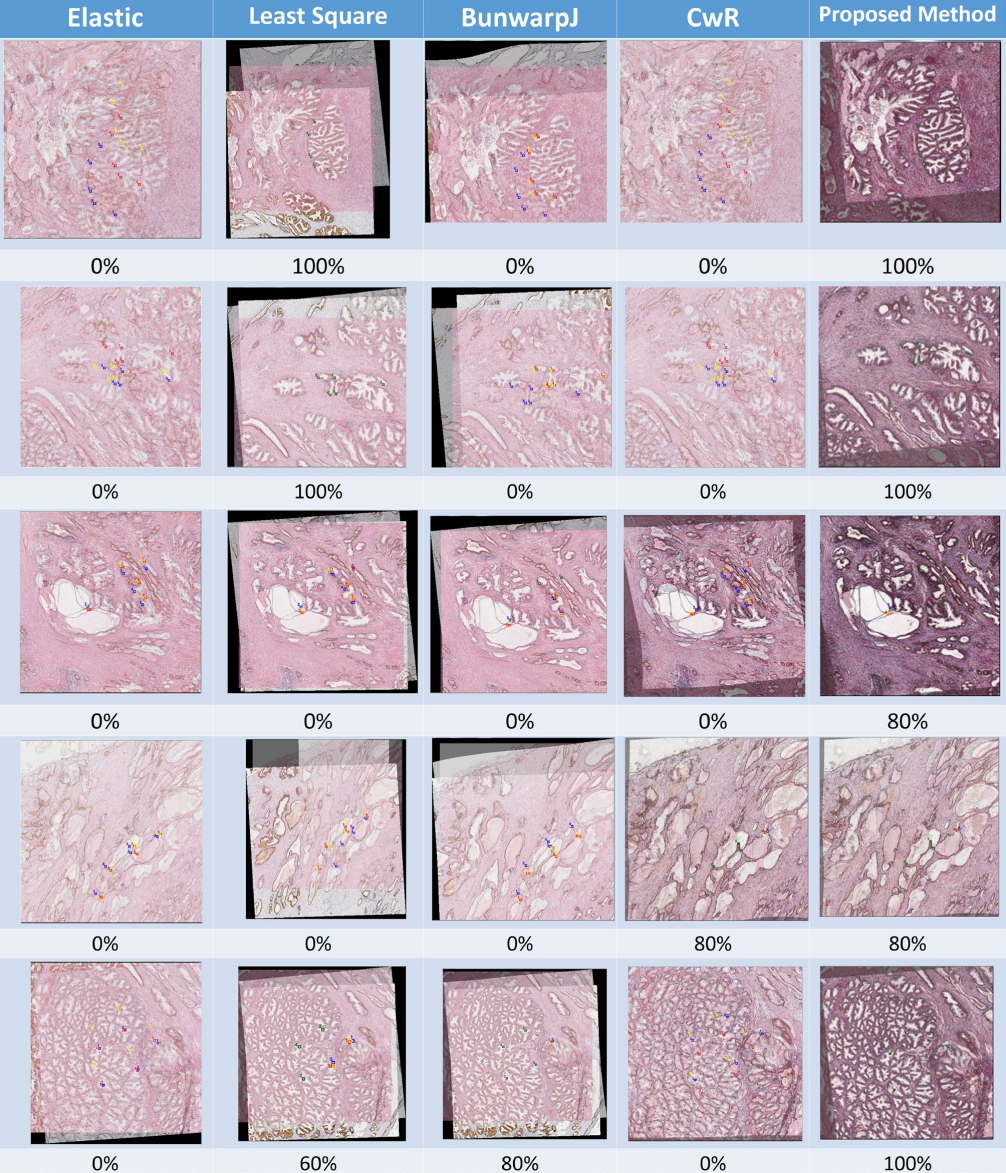
Registration results of the proposed method and benchmark approaches^7–10^ in triple-staining prostate cancer samples. The blue rectangles represent the locations of the selected landmarks defined by experienced pathologists in the target image; the red boxes represent mismatches of corresponding landmarks in the transformed source image (IHC1); the yellow boxes represent mismatches of corresponding landmarks in the transformed source image (IHC2); the green boxes represent matches of corresponding landmarks in the transformed source image.

Regarding the evaluation method to measure the registration accuracy, as pointed out in the previous studies^45,46^, the traditional evaluation approach based on sum of squared differences of image intensities between the target and the transformed source could be inaccurate in biological image applications as the pixel intensity in the target and the intensity of the accurately registered pixel in the transformed source are generally different due to stain variation. As a result, the quantitative evaluation approach of the previous works^45,46^ is adopted where five corresponding landmarks between target images and associated transformed source images by each registration method were firstly manually marked by experienced pathologists, and an automatic matching system is applied to compare the coordinates of the corresponding manual annotations, producing registration accuracy scores for each image pair based on the matching successful rate over the corresponding annotations (within a five pixel distance). The final registration accuracy score of each registration method is computed using the averaged accuracy over all image pairs as shown in Fig. 3.

#### Quantitative evaluation on the triple-staining prostate cancer dataset

**Figure 4:**
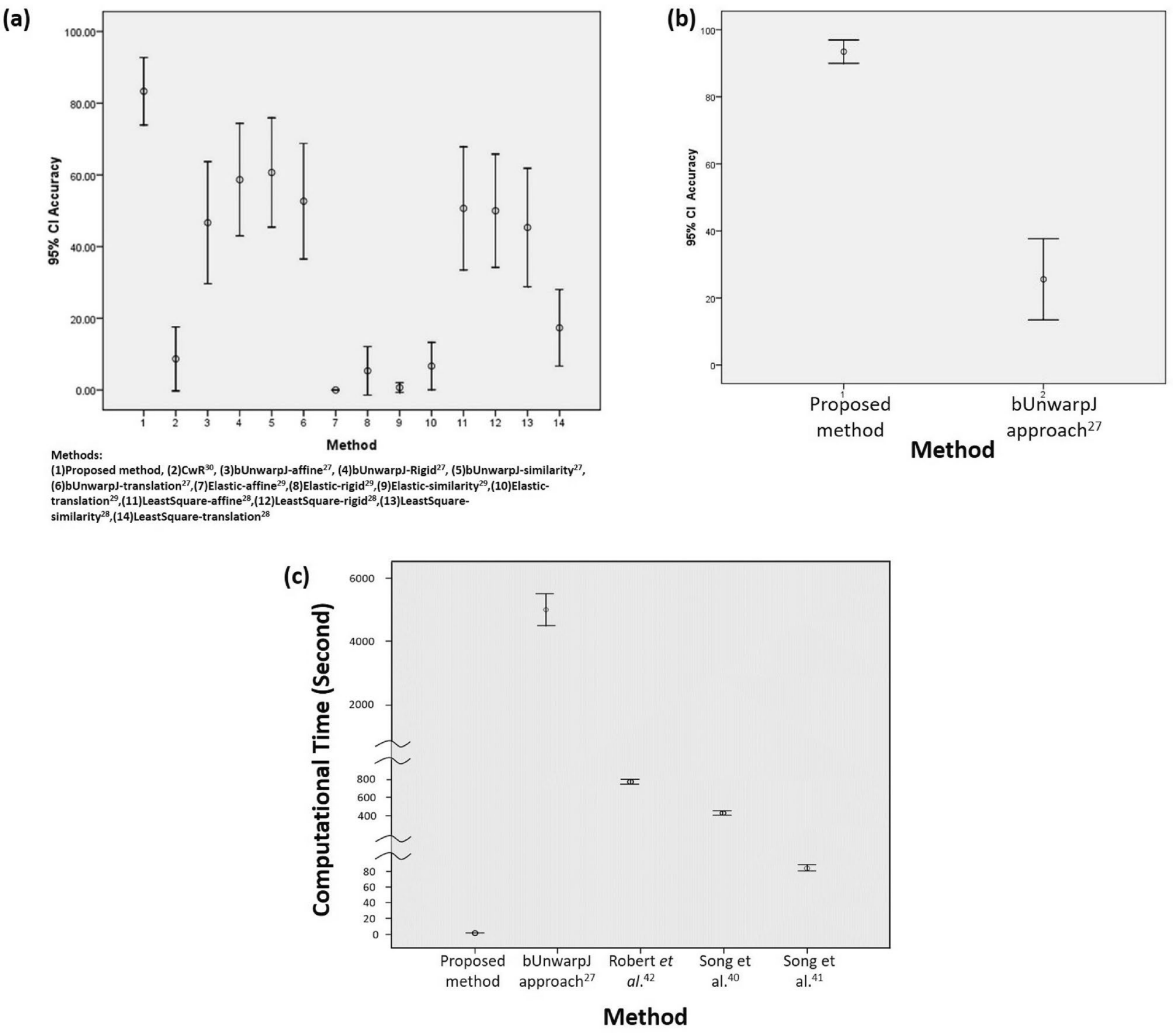
(**a**) Evaluation results on the triple staining prostate cancer slides by the proposed method and benchmark approaches^7–10^. (**b**) Evaluation results on the dual staining breast cancer slides by the proposed method and the best performed benchmark approach in the first experiment^7^. (**c**) Run time analysis in cross-staining WSI registration of the proposed method and benchmark approaches^7,27,28,47^.

Figure 4a presents the evaluation results of registration accuracy on triple-staining prostate cancer tissue images by the proposed method and four benchmark approaches, including bUnwarpJ^7^, Leastsquares^8^, Elastic^9^ and CwR^10^. In addition, four transformation models, including affine, rigid, similarity and translation, are applied in testing of the benchmark approaches except for the CwR^10^. As shown in Fig. 4, the proposed method consistently performs well and significantly outperforms benchmark methods. Detailed quantitative results could be found in Table 2. In addition, we further conduct the statistical analysis, and the result shows that the proposed method is significantly better than the benchmark methods ($p<0.01$) for triple-staining prostate cancer tissue image registration. Figure 3 shows some sample results of registration outputs of the proposed method and benchmark approaches.

**Table 2:** Quantitative evaluation results on the triple staining prostate cancer dataset.

95% confidence interval for mean														
	A	B	C	D	E	F	G	H	I	J	K	L	M	N
Mean	83.33	8.67	46.67	58.67	60.67	52.67	0.00	5.33	.67	6.67	50.67	50.00	45.33	17.33
Std. deviation	6.74	23.89	45.59	42.00	10.92	43.15	0.00	18.14	3.65	17.68	46.01	42.26	44.24	28.64
Std.error	4.61	4.36	8.32	7.67	7.46	7.88	0.00	3.31	0.67	3.23	8.40	7.72	8.08	5.23
Lower bound	73.91	0.00	29.64	42.99	45.41	36.56	0.00	0.00	0.00	0.06	33.49	34.22	28.82	6.64
Upper bound	92.76	17.56	63.69	74.35	75.92	68.78	0.00	12.11	2.03	13.27	67.85	65.78	61.85	28.03

LSD test analysis of the proposed method and benchmark approaches														
	A-B	A-C	A-D	A-E	A-F	A-G	A-H	A-I	A-J	A-K	A-L	A-M	A-N	
Mean difference	74.67*	36.67*	24.67*	22.67*	30.67*	83.33*	78*	82.67*	67.67*	32.67*	33.33*	38*	66.00*	
Std.error	8.71	8.71	8.71	8.71	8.71	8.71	8.71	8.71	8.71	8.71	8.71	8.71	8.71	
*P*	<.01	<.01	<.01	<.01	<.01	<.01	<.01	<.01	<.01	<.01	<.01	<.01	<.01	
Lower bound	57.55	19.55	7.55	5.55	13.55	66.22	60.89	65.55	59.55	15.55	16.22	20.89	48.89	
Upper bound	91.78	53.78	41.78	39.78	47.78	100.45	95.11	99.78	93.78	49.78	50.45	55.11	83.11	

Methods: (A) Proposed method, (B) CwR^10^, (C) bUnwarpJ-affine^7^, (D) bUnwarpJ-rigid^7^, (E) bUnwarpJ-similarity^7^, (F) bUnwarpJ-translation^7^, (G) Elastic-affine^9^, (H) Elastic rigid^9^, (I) Elastic-similarity^9^, (J) Elastic-translation^9^, (K) LeastSquare-affine^8^, (L) LeastSquare-rigid^8^, (M) LeastSquare-similarity^8^, and (N) LeastSquare-translation^8^

The proposed method is significantly better than the benchmark approaches ($p<0.01$)

#### Quantitative evaluation on the dual-staining breast cancer dataset

Figure 4b compares the evaluation results on dual-staining breast cancer tissue images by the proposed method and the best benchmark approach performed in the first experiment, i.e, bUnwarpJ^7^. The proposed method achieves high averaged registration accuracy (93.49%) while the bUnwarpJ approach^7^ obtains averaged accuracy only 25.58%. Detailed quantitative results could be found in Table 3. Using SPSS software^44^, the result shows that the proposed method significantly performs better than bUnwarpJ^7^ ($p<0.001$).

**Table 3:** Results on the breast cancer dataset in comparison of the proposed method and the best performed benchmark approach in the first experiment.

95% confidence interval for mean					
Method	Mean	Std. deviation	Std.error	Lower bound	Upper bound
Proposed method	93.15	4.55	3.13	86.8	99.51
bUnwarpJ^7^	25.58	39.35	6	13.47	37.69

LSD test analysis of the proposed method and benchmark approaches					
	Mean difference	Std. error	*P*	Lower bound	Upper bound
Proposed method—bUnwarpJ^7^	67.91*	6.53	<.001	54.74	81.08

*The proposed method is significantly better than the benchmark approach ($p<0.001$)

#### Run time analysis

For run time analysis, the breast cancer dataset is used, and the computational time of WSI registration of the proposed method is compared with the best benchmark approach performed in the first experiment, i.e. bUnwarpJ approach^7^, and three additional benchmark approaches, including Roberts et al.^27^, Song et al.^28^ and Lotz et al.^29^. In testing of WSI registration, as the system of bUnwarpJ approach^7^ tends to fail due to out of memory, we therefore perform a regression analysis to estimate the computing time for WSI registration using least squares. Detailed quantitative results could be found in Table 4. The proposed method only takes 4.34 s per WSI registration on average. In comparison, bUnwarpJ approach^7^ costs 55162.4 s (15.3 hours) per WSI registration on average. Roberts et al.^27^ and Song et al.^28^ take more than 400 s (6.67 minutes) per WSI registration on average, and Lotz et al.^29^ costs 76.42 s per WSI registration on average. Figure 4c displays the distributions of the WSI registration computational time by individual methods, showing that the proposed method takes much less time than all benchmark approaches^7,27–29^. By conducting LSD test, the proposed method is significantly faster than the benchmark approaches^7,27–29^ with $p<0.001$ in WSI registration. In addition, the proposed method is able to finish WSI registration within 1 s for 30.23% WSI pairs of the experimental dataset. A demonstration of real time cross staining WSI registration by the proposed method is presented in the supplementary video. (https://www.youtube.com/watch?v=0Uc6-s_ClIg&ab_channel=ProfChing-WeiWang).

**Table 4:** Run time analysis in cross-staining WSI registration.

95% confidence interval for mean (Unit: second)					
Method	Mean	Std. deviation	Std.error	Lower bound	Upper bound
Proposed method	4.34	2.45	0.38	3.57	5.11
bUnwarpJ^7^	55162.40	3784.17	577.08	53997.81	56327.00
Robert et al.^27^	799.89	86.79	13.23	773.17	826.59
Song et al.^28^	484.33	152.47	23.25	437.40	531.25
Lotz et al.^29^	76.42	6.98	1.06	74.27	78.57

LSD test analysis of the proposed method and benchmark approaches					
Method	Mean difference	Std. error	*P*	Lower bound	Upper bound
Proposed method—bUnwarpJ^7^	− 55158.06*	576.74	< 0.001	− 56321.98	− 53994.15
Proposed method—Robert et al.^27^	− 795.54*	13.26	< 0.001	− 822.29	− 768.89
Proposed method—Song et al.^28^	− 479.98*	23.27	< 0.001	− 526.94	− 433.02
Proposed method—Lotz et al.^29^	− 70.33*	1.29	< 0.001	− 72.93	− 67.73

*The proposed method performs significantly faster than the benchmark approaches ($p<0.001$)

## Discussion

Cross-staining alignment and joint analysis of multiple IHC and H&E slides are important for disease diagnosis, drug development and biological research, enabling simultaneous assessment of multiple kinds of protein expressions for tissue types of interests at single-cell resolution. Taking the diagnosis of invasive prostate carcinoma case, in clinical practice serial sections of prostate cancer donor are stained with three different stains, including HMCK, CK18 and H&E as shown in Fig. 1b. To identify cancerous tissues, medical doctors have to compare those three different slides, which is time-consuming, subject and difficult to quantify tumors. Automatic cross-staining biological image alignment helps medical doctor to identify cancerous tissues, quantify the amount of cancerous tissues and determine the cancer stage. Figure 1b shows triple-staining alignment on prostate cancer tissue sample results performed by the proposed method.

In prognosis of breast cancer patients, metastatic foci to lymph nodes have been classified into macrometastases (metastatic size greater than 2.0 mm), micrometastases (metastatic size greater than 0.2 mm, but none greater than 2.0 mm), and isolated tumour cells (ITCs, metastatic size no greater than 0.2 mm). Currently, the status of lymph nodes is routinely determined by the examination of H&E slides. Immunohistochemical staining for cytokeratin on lymph node section may be applied to rule out ITCs, which are mostly detected by IHC due to their tiny size. Despite the clinical significance of ITCs or micrometastases is debated, the results of the MIRROR trial emphasized the need for additional therapy in cases of invasive breast carcinoma with ITCs or micrometastases^40–43^, supporting that the identification of ITCs or micrometastases is currently clinically needed. However, because IHC for cytokeratin is not routinely performed on every axillary lymph node of breast cancer patients, it is possible that ITCs may run undetected, particularly in cases with many axillary lymph nodes, and result in under-treatment of the patients. Furthermore, the routine examination of H&E slides of lymph nodes, particularly for axillary lymph node dissection which may include many lymph nodes, is time-consuming and pathologists may potentially run the risk of missing tiny metastatic foci. In this study, automatic dual-staining WSI alignment of H&E and IHC CK(AE1/AE3) slides is performed, which help medical doctors to screen all the lymph nodes sections and identify potential tiny metastatic foci as shown in Fig. 1b. Due to the budge limitation, immunohistochemistry for CK can be performed on two sentinel lymph nodes (LN), usually sentinel LN1 and LN2. However, in clinical practice, we are very frequently encountering more than two sentinel LNs plus axillary lymph node dissections, which may include many lymph nodes. This method can help identifying potential micrometastasis and ITCs that may go unnoticed by the pathologists in lymph nodes sections without immunohistochemistry for CK.

In the experiments of triple-staining alignment on 90 prostate cancer tissue images, the proposed method achieves the triple-staining registration accuracy 0.833±0.0674, while four benchmark approaches^7–10^ perform poor, obtaining the averaged accuracy no higher than 0.60. In statistical analysis, the proposed method is significantly better than all benchmark approaches^7–10^ ($p<0.01$) for cross-staining microscopic image registration. In alignment of breast cancer tissue images evaluation, we compare the proposed method and the best benchmark approach performed in the triple-staining alignment of prostate cancer tissue images, i.e bUnwarpJ^7^ on 86 breast cancer tissue images. In evaluation, the proposed method achieves high image registration accuracy 0.931±0.0455. In comparison, the benchmark method - bUnwarpJ approach only obtains averaged accuracy 0.255. In statistical analysis, the proposed method significantly outperforms bUnwarpJ approach ($p<0.001$) for dual-staining alignment of breast cancer tissue images.

For run time analysis, the proposed method takes only 4.34 s per WSI registration on average. In comparison, the benchmark approaches^7,27–29^ spend more than 70 s per WSI registration on average. In addition, for 30.23% data, i.e. 13 out of 43 WSI pairs of breast cancer data, the proposed method takes less than 1 s for WSI registration. In this study, we present a real-time, fully automatic and robust cross-staining registration system for aligning multiple IHC slides and histopathological H&E slides to assist assessment of tissue morphology and various protein expressions together, using two different digital scanners, i.e. Leica and 3DHISTECH, on two different cancer samples, i.e. breast cancer and prostate cancer, and from two different hospitals. The proposed method is not limited for cross-staining analysis but could also be used for single-staining serial section comparison.

## Methods

**Figure 5:**
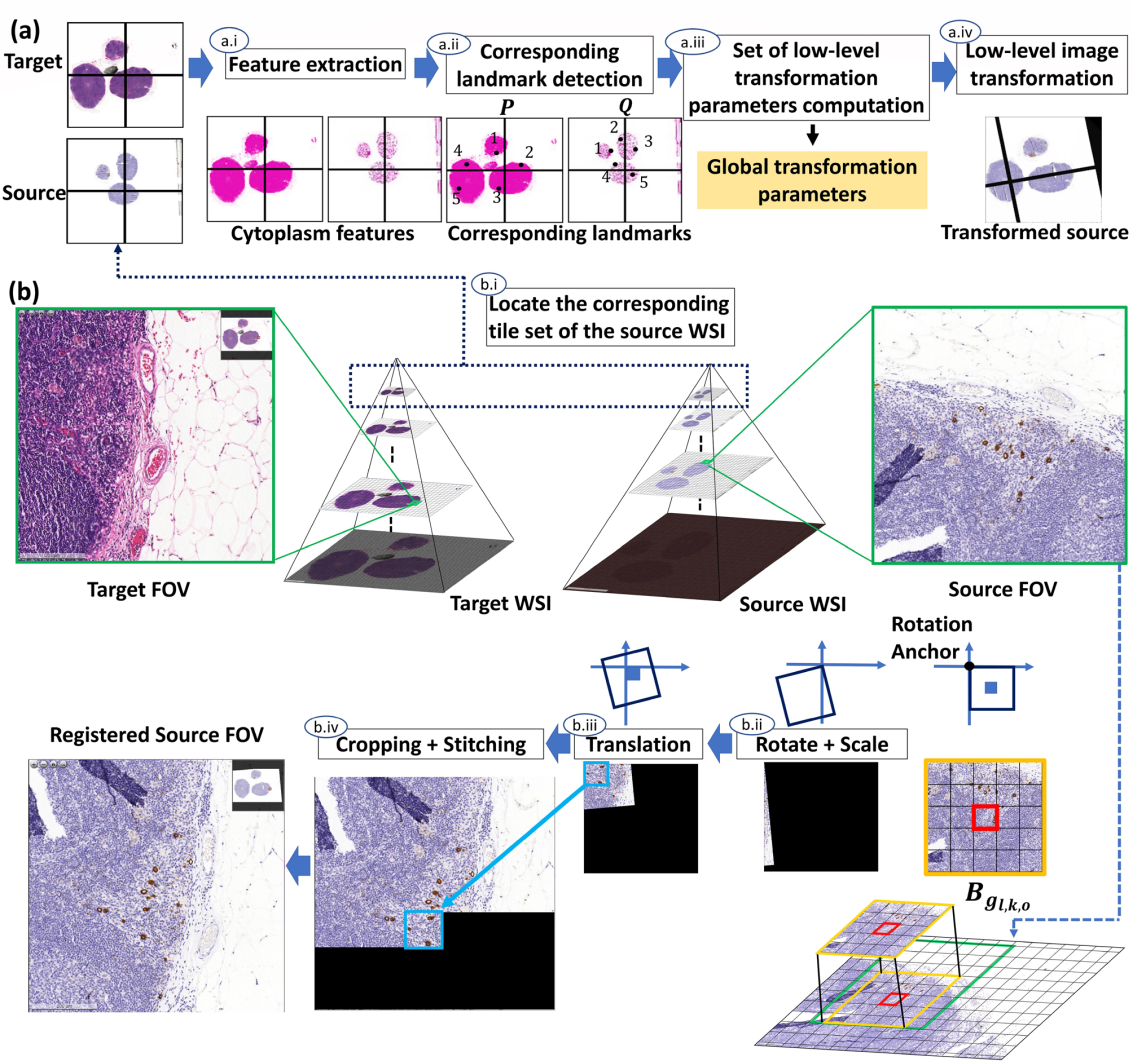
Flowchart of the proposed real time interactive cross staining WSI alignment framework, consisting of (**a**) a rapid global image registration module and (b) a real time propagated multi-level image registration. For (a) rapid global registration, (a.i) a stain separation model is built to extract the cytoplasm features; (a.ii) Corresponding landmarks are detected using the cytoplasm features; (a.iii) Based on the corresponding landmarks, global transformation parameters are generated; (a.iv) A global image registration result is obtained by applying the global transformation parameters onto the low-level image. For (**b**) multi-level image registration, a tile set of the source WSI corresponding to the Field of View (FOV) of the target WSI are fetched using the global transformation parameters. (b.i) The fetched tile set as the source FOV highlighted in green is used as input, and each tile is selected as the center, which is colored in red, to obtain an enlarged area highlighted in orange. After that, each enlarged area is scaled and then rotated clockwise by the rotation anchor as center. (b.ii) The top left tile highlighted in blue is translated; (b.iii) The tile is then cropped and stitched into the registration output image; (b.iv) The registered source FOV is produced.

In this study, we present a real-time and fully automatic coarse to fine propagated tile-based registration framework for cross-staining WSI alignment. The proposed framework consists of two main parts, including a rapid global registration module (“Rapid global image registration” section ) and a propagated real time multi-level image registration module (“Propagated multi-level image registration” section). Fig. 5 presents the flowchart of the proposed method. For the rapid global registration module as illustrated in Fig. 5a., firstly, cytoplasm features of low-level images are extracted using color deconvolution (“Cytoplasm feature extraction model” section); secondly, corresponding landmarks between the target and the source image are detected (“Corresponding landmark detection” section) and then used to compute the global transformation parameters (“Computation of global transformation parameters” section); thirdly, the low-level source image are aligned to the target by using the global transformation parameters (“Global image transformation” section). For the second part of the proposed method, i.e. the real time propagated multi-level image registration module as illustrated in Fig. 5b), firstly, the tile set of the source WSI, corresponding to the Field of View (FOV) of the target WSI, are extracted using the transformation parameters; secondly, the source FOV is aligned to the target FOV by the proposed real time patch-wise registration (“Propagated multi-level image registration” section).

All methods were carried out in accordance with relevant guidelines and regulations. The experimental protocols were approved by the research ethics committees of the National Taiwan University Hospital (with ethical approval number: NTUH-REC 201810082RINB) and the Tri-Service General Hospital, Taipei, Taiwan ((with ethical approval number: TSGHIRB1-107-05-171). Informed patient consent forms were formally waived by the research ethics committees of the National Taiwan University Hospital and Tri-Service General Hospital, and the data were de-identified and used for a retrospective study without impacting patient care.

### Rapid global image registration

Let $$\{\ {\mathcal {J}}^{\xi } \}\ $$ be a pair of digital WSI, containing a target WSI ${\mathcal {J}}^t$ and a source WSI ${\mathcal {J}}^s$ for alignment where $\xi =t$ represents the target, and $\xi =s$ represents the source, respectively. We formulate a set of digital WSIs $$\{\ {\mathcal {J}}^{\xi } \}\ $$ into multi-level pyramid tile-based data structure $$\{\ I^{\xi }_{l,i,j} \}\ $$ with multiple layers from low to high magnification, where *i* and *j* represent the column and row number of a tile in level *l*, respectively, and the unit of the tile size is $m \times m$, where $m=256$ in this study. Low-level target and source tile-images $I^{t}_{low}, I^{s}_{low}$ are extracted from target and source WSIs at level $l_{low}$, and $l_{low} = \sum {I_{l}} <=(2m)^{2}$. $I^{t}_{low}$ and $I^{s}_{low}$ are used for the rapid global image registration process.

To obtain the global transformation parameters and low-level image registration result, low-level image registration process comprises four parts as shown in Fig. 5a. (1) Using colour deconvolution technique with an orthonormal transformation matrix, stain separation is performed on the low-level target and source tile-images to extract the cytoplasm features of the target and source. (2) Corresponding landmark detection is applied to the cytoplasm features images obtained from the previous step for establishing pairwise correspondences between the target and the source. (3) By aligning pairwise correspondences, global transformation parameters (rotation matrix, scaling factor and translation vector) are generated based on the corresponding landmarks detection. (4) The low-level source image is aligned to the target using the transformation parameters obtained from step 3, producing a low-level registered source. The low-level global transformation parameters is then used for real-time interactive FOV localization and real-time multi-level image registration.

#### Cytoplasm feature extraction model

In RGB colour, each colour can be represented as $\vec {c} \equiv (c_{1}, c_{2}, c_{3})$. To model the colour in an image as the vector addition of a desired (D), undesired (U) and a background (P), a new vector model is defined as follow:

1 $$\begin{aligned} \vec {u}= & {} \overrightarrow{PU} \end{aligned}$$

2 $$\begin{aligned} \vec {d}= & {} \overrightarrow{PD} \end{aligned}$$

3 $$\begin{aligned} \vec {n}= & {} \vec {u} \times \vec {d} \end{aligned}$$

Then, colour $\vec {c}$ is transformed to the new unit vector $\vec {c} = u.\vec {u} + d.\vec {d} + n.\vec {n} + \vec {p}$. By setting $u = 0$, the undesired component is removed, generating new colour $\vec {c_{\prime}} = d.\vec {d} + n.\vec {n} + \vec {p}$. In three channels image, a colour system is described as a matrix form $\mu$ with every row representing a specific stain and every column representing the Optical Density (OD) as detected by $c_1$, $c_2$ and $c_3$ for each stain, respectively.

4 $$\begin{aligned} \mu = \begin{bmatrix} c_{11} &{} c_{12} &{} c_{13}\\ c_{21} &{} c_{22} &{} c_{23}\\ c_{31} &{} c_{32} &{} c_{33} \end{bmatrix} \end{aligned}$$

In this study, a normalized OD matrix, ${\widehat{\mu }}$, to describe the colour system for orthonormal transformation is defined as follows:

5 $$\begin{aligned} {\widehat{\mu }} = \begin{bmatrix} 0.6442 &{} 0.7166 &{} 0.2668\\ 0.0928 &{} 0.9541 &{} 0.2831\\ 0 &{} 0 &{} 0 \end{bmatrix} \end{aligned}$$

Let $G = [g_0, g_1, g_2]$ be denoted as a $3 \times 1$ vector for the amount of stains at the specific pixel, where $g_0$, $g_1$ and $g_2$ represent the first, second and third channel, respectively. The OD levels at individual pixels could be formulated as $O = G{\widehat{\mu }}$. Therefore, multiplication of the OD image with the inverse of the OD matrix results in orthogonal representation of the stains forming the image $G = {\widehat{\mu }}^{-1}O$. Then, the cytoplasm features, $g^{1}_{0}$ and $g^{2}_{0}$, of the target image and the source image are extracted for further corresponding landmark detection.

#### Corresponding landmark detection

Given a pair of cytoplasm features, $g^{1}_{0}$ and $g^{2}_{0}$, a set of possible transformations ${\mathcal {T}}$ between $g_{0}^{1}$ and $g_{0}^{2}$, and a mapping function $H_\tau (g_{0}^{2})$ , we aim to obtain the optimal transformation $\tau ^{\prime} = \arg \min _{\tau \in {\mathcal {T}}} ||H_\tau (g_{0}^{2}) - g_{0}^{1}||^{2}$. The optimal transformation $\tau ^{\prime}$ can be achieved using the shortest distance of the transformation invariant $\theta (g_{0}^{1}, g_{0}^{2}) = ||H_{\tau ^{\prime}}(g_{0}^{2}) - g_{0}^{1}||^{2}$, which corresponds to the Euclidean distance in $${\L }{^2} = \{\ \Upsilon : \Re \rightarrow \Re : \int _{-\infty }^{\infty }|\Upsilon (\nu )^{2}|d\nu <\infty \}\ $$. However, the optimal transformation $\tau ^{\prime}$ and the shortest distance $\theta (g_{0}^{1}, g_{0}^{2})$ are not easy to achieve as the objective function, because it is non-convex and local minima trapped solution might occur. To cope with the aforementioned issues, we apply a set of geometric features $\Gamma = \{\ \psi _{\gamma ^{1}} : \gamma ^{1} \in {\mathcal {T}}_{\theta } \}\ \subset \L ^{2}$, which is constructed by transforming a generating function $\psi \in \L ^{2}$, where ${\mathcal {T}}_{\theta } \subset {\mathcal {T}}$ represents a finite discretization of the transformations ${\mathcal {T}}$ and $\psi _{\gamma ^{1}} = U_{\gamma ^{1}}(\psi )$ represents the transformation of the generating function $\psi$ by $\gamma ^{1}$.

Let $P={ \{\ p_{k}\}\ }_{k=0}^{K}$ and $Q={ \{\ q_{k}\}\ }_{k=0}^{K}$ be two sets of pairwise correspondences between $g_{0}^{1}$ and $g_{0}^{2}$ in $\Gamma$, respectively,

6 $$\begin{aligned} P= & {} \sum _{k=0}^{K} \alpha _{k}\psi _{\gamma ^{1}_{k}} \end{aligned}$$

7 $$\begin{aligned} Q= & {} \sum _{k=0}^{K} \beta _{k}\psi _{\gamma ^{2}_{k}} \end{aligned}$$

where $\alpha _{k}$ and $\beta _{k}$ are not negative coefficients.

Two sets of pairwise correspondences *P* and *Q* are obtained by the following steps: (1) key point sets $E^{1}$ and $E^{2}$ are detected by applying Difference of Gaussian (DoG) detector^48^, (2) the corresponding landmarks *P* and *Q* are selected as geometric consensus between keypoints $E^{1}$ and $E^{2}$ by applying Random Sample Consensus (RANSAC)^49^. The corresponding feature detection *P* and *Q* are then used to compute a set of global transformation parameters.

#### Computation of global transformation parameters

By aligning two sets of pairwise correspondences, $P = { \{\ p_{k}\}\ }_{k=0}^{K}$ and $Q = { \{\ q_{k}\}\ }_{k=0}^{K}$, the task is to compute a set of global transformation parameters rapidly at low resolution level, including scaling factor *S*, the rotation matrix *R* and the translation vector *T*. Firstly, to computes the scaling factor *S*, the centroids of each set are calculated, then both of the centroids are translated to its origin (the centred vectors). Let ${\overline{p}} = \frac{\sum _{k=0}^{K}p_{k}}{K}$ and ${\overline{q}} = \frac{\sum _{k=0}^{K}q_{k}}{K}$ be the centroids of each set, and $p_{k}^{\prime} = p_{k}-{\overline{p}}$ and $q_{k}^{\prime} = q_{k}-{\overline{q}}$ be the centred vectors, the scaling factor *S* can be computed as follows:

8 $$\begin{aligned} S = \sqrt{\sum _{k=0}^{K}\frac{\sigma (p_{k}^{\prime})}{\sigma (q_{k}^{\prime})}} \end{aligned}$$

where $\sigma$ represents the variance.

Next, $d \times d$ covariance matrix *C* is computed: $C=\sum _{k=0}^{K}(q_{k}^{\prime} \times {p_{k}^{\prime}}^{tr})$, and *tr* is matrix transpose operator. Then using Jacobi Singular Value Decomposition (SVD) algorithm^50^ $\delta$, the SVD matrix *X* is computed: $X=\delta (C)$. To obtain the rotation matrix *R*, the SVD matrix *X* is decomposed, $UZV=X$, where *U*, *V* are rotation matrices and *Z* is a diagonal matrix. The rotation matrix *R* can be computed by the following expression:

9 $$\begin{aligned} R = \begin{bmatrix} R_{00} &{} R_{01} &{} R_{02}\\ R_{10} &{} R_{11} &{} R_{12}\\ R_{20} &{} R_{21} &{} R_{22} \end{bmatrix} = {\left\{ \begin{array}{ll} VEU^{tr}, &{} det(VU^{tr}) \\ VU^{tr}, &{} otherwise \end{array}\right. } \end{aligned}$$

where $$E= \begin{bmatrix} 1 &{} 0 &{} 0\\ 0 &{} 1 &{} 0\\ 0 &{} 0 &{} 1 \end{bmatrix}$$ is a $3 \times 3$ diagonal identity matrix.

After computing scaling factor *S* and rotation matrix *R*, the translation vector *T* can be computed by:

10 $$\begin{aligned} T = ({\overline{p}} - RS{\overline{q}}) \end{aligned}$$

*S*, *R* and *T* are then used for aligning low-level source image $I^{s}_{low}$ to the target $I^{t}_{low}$, real-time interactive FOV localization and real-time multi-level image registration processes.

#### Global image transformation

After obtaining a set of transformation parameters from the previous section, next step is to align the low-level source image $I^{s}_{low,(x,y,w,h)}$ to the target image and to produce a low-level registered-source $I^{s'}_{low,(x^{\prime},y^{\prime},w,h)}$, where (*x*, *y*) is the coordinate of $I^{s}_{low,(x,y,w,h)}$ and $(x^{\prime},y^{\prime})$ is the coordinate of $I^{s'}_{low,(x^{\prime},y^{\prime},w,h)}$. The mapping relationship is formulated as follows.

11 $$\begin{aligned} \begin{bmatrix} y^{\prime}\\ x^{\prime}\\ 0 \end{bmatrix} = \Xi (R,S,T,x,y) = {\left( RS\begin{bmatrix} y\\ x\\ 0 \end{bmatrix} + T \right) } \end{aligned}$$

### Propagated multi-level image registration

Real-time interactive FOV localization module is devised to locate and fetch the associated tile set of the source WSI corresponding to the FOV of the target WSI as shown in Fig. 5b. Let $F^{t} = I^{t}_{l,(u^t,v^t,w^t,h^t)}$ be the FOV of the target WSI, where $(u^t,v^t)$ is the global coordinate of $F^{t}$; $n_1 \times n_2$ is the number of tiles containing $F^t$. Firstly, the global coordinate $(u^s,v^s)$ of the top left tile of the FOV in the source WSI corresponding to the target FOV $F^t$ is computed,

12 $$\begin{aligned} \begin{bmatrix} v^s\\ u^s\\ 0 \end{bmatrix} = \left( (SR)^{-1} \left( \begin{bmatrix} v^t\\ u^t\\ 0 \end{bmatrix}-2^{\triangle }T \right) ^{}\right) ^{} \end{aligned}$$

where $\triangle = l-l_{low}$ is used for level calibration.

Secondly, the associated tile set of the source WSI corresponding to $F^t$ is fetched: $G:{\{\ g_{l,i,j} \}\ }_{\{\ i=a,\ldots ,a+n_1,j=b,\ldots ,b+n_2 \}\ }$. The associated tile set is then used to compute registration outputs. Next, transformation is applied to each enlarged area of every tile $g_{l,i,j}$ in the fetched tile set, producing a transformed enlarged area. Afterwards, the top left tile of each transformed enlarged area is selected and then integrated into an image as the registration output, i.e. the corresponding FOV of the source WSI (see Fig. 5b). Detailed registration processes of the proposed method are described as follows.

Let $B_{g_{l,k,o},(x,y,w,h)} = { \{\ b_{l,i,j} \}\ }_{ i=k-\lfloor {\frac{q}{2}}\rfloor ,\ldots ,k+\lfloor {\frac{q}{2}}\rfloor ,j=o-\lfloor {\frac{q}{2}}\rfloor ,\ldots ,o+\lfloor {\frac{q}{2}}\rfloor }$ be an enlarged area containing $q \times q$ tiles of each $g_{l,i,j}$ as the center tile, where $k=a,\ldots ,a+n_{1}$, $o=b,\ldots ,b+n_2$, $x = (k-\lfloor {\frac{q}{2}}\rfloor )m$, $y = (o-\lfloor {\frac{q}{2}}\rfloor )m$, $w \times h =(qm)^2$, and $q = 5$ in this study. Firstly, the translation factor is computed for each enlarged area $B_{g_{l,k,o},(x,y,w,h)}$ at level *l*:

13 $$\begin{aligned} T_{g_{l,k,o}}=\left( -mR\begin{bmatrix} (o-\lfloor {\frac{q}{2}}\rfloor )\\ (k-\lfloor {\frac{q}{2}}\rfloor )\\ 0 \end{bmatrix}\right) \end{aligned}$$

Secondly, each enlarged area $B_{g_{l,k,o},(x,y,w,h)}$ is transformed in parallel by the following formula:

14 $$\begin{aligned} B_{gl,k,o}= & {} { \{\ b_{l,i,j}\}\ }_{i=k-\lfloor {\frac{q}{2}}\rfloor ,\ldots ,k+\lfloor {\frac{q}{2}}\rfloor ,j=o-\lfloor {\frac{q}{2}}\rfloor ,\ldots ,o+\lfloor {\frac{q}{2}}\rfloor }\nonumber \\= & {} {\{\ I^{t}_{l,(x,y,qm,qm)}\}\ }|_{(k-\lfloor {\frac{q}{2}}\rfloor )m\le x\le (k+\lfloor {\frac{q}{2}}\rfloor )m,(o-\lfloor {\frac{q}{2}}\rfloor )m\le y\le (o+\lfloor {\frac{q}{2}}\rfloor )m} \end{aligned}$$

15 $$\begin{aligned} T^{\prime}_{g_{l,k,o}}= & {} R \begin{bmatrix} -(m-2\lambda _x)*\lfloor {\frac{q}{2}}\rfloor \\ -(m-2\lambda _y)*\lfloor {\frac{q}{2}}\rfloor \\ 0 \end{bmatrix} +T_{g_{l,k,o}} \end{aligned}$$

16 $$\begin{aligned} B^{\prime}_{g_{l,k,o},(x^{\prime},y^{\prime},w,h)}=\, & {} SRB_{g_{l,k,o}}+T^{\prime}_{g_{l,k,o}} \nonumber \\=\, & {} \{I^{\prime}_{l,(x^{\prime},y^{\prime},qm,qm)}\}|_{(k-\lfloor {\frac{q}{2}}\rfloor )m\le x\le (k+\lfloor {\frac{q}{2}}\rfloor )m,(o-\lfloor {\frac{q}{2}}\rfloor )m\le y\le (o+\lfloor {\frac{q}{2}}\rfloor )m} \end{aligned}$$

where $B^{\prime}_{g_{l,k,o},(x^{\prime},y^{\prime},w,h)}$ represents a set of transformed enlarged areas, $\lambda _x$ and $\lambda _y$ means the overlapping part between each tiles.

Thirdly, a tile from each transformed enlarged area $B^{\prime}_{g_{l,k,o},(x^{\prime},y^{\prime},w,h)}$ is selected as the registration output tile: $z_{g_{l,k,o}} = I^{\prime}_{l,((k-\lfloor {\frac{q}{2}}\rfloor )m,(o-\lfloor {\frac{q}{2}}\rfloor )m,qm,qm)}$. Finally, $n_1 \times n_2$ tiles are integrated as the corresponding FOV of the source WSI: $$Z =\{\ z_{g_{l,k,o}} \}\ $$.

## Supplementary Video

An online web-based system of the proposed method has been created for live demonstration of real time cross staining alignment of whole slide images. Please see the supplementary video at https://www.youtube.com/watch?v=0Uc6-s_ClIg&ab_channel=ProfChing-WeiWang.
